# Reactive and Proactive Adaptation of Cognitive and Motor Neural Signals during Performance of a Stop-Change Task

**DOI:** 10.3390/brainsci11050617

**Published:** 2021-05-11

**Authors:** Adam T. Brockett, Matthew R. Roesch

**Affiliations:** 1 Department of Psychology, University of Maryland, College Park, MD 20742, USA; mroesch@umd.edu; 2 Program in Neuroscience and Cognitive Science, University of Maryland, College Park, MD 20742, USA

**Keywords:** inhibitory control, executive function, action selection, decision making

## Abstract

The ability to inhibit or suppress unwanted or inappropriate actions, is an essential component of executive function and cognitive health. The immense selective pressure placed on maintaining inhibitory control processes is exemplified by the relatively small number of instances in which these systems completely fail in the average person’s daily life. Although mistakes and errors do inevitably occur, inhibitory control systems not only ensure that this number is low, but have also adapted behavioral strategies to minimize future failures. The ability of our brains to adapt our behavior and appropriately engage proper motor responses is traditionally depicted as the primary domain of frontal brain areas, despite evidence to the fact that numerous other brain areas contribute. Using the stop-signal task as a common ground for comparison, we review a large body of literature investigating inhibitory control processes across frontal, temporal, and midbrain structures, focusing on our recent work in rodents, in an effort to understand how the brain biases action selection and adapts to the experience of conflict.

## 1. Introduction

Imagine for a second that you are driving a car, and have just pulled up at a red light. To your left is a turn lane that has its own separate light, and to your right another lane that, like yours, will continue straight ahead through the intersection. Now that you have imagined this scenario, have you ever found yourself suddenly slamming on the brake after letting off it for a second, thinking that the green left turn arrow signal for the lane next to yours, was actually instructing *you* to go as well? Hopefully, we’re not alone in this experience.

In truth, I know we are not alone, because just recently, to my amazement, I witnessed a car in the lane to my right (i.e., two full lanes from the turn lane which had just received a green arrow) lurch fully into the intersection, slam on their brakes, and then speed forward to avoid being hit by turning traffic coming from the opposite direction. While not meaning to minimize the danger this motorist caused, I instinctively said out loud “well someone’s anterior cingulate cortex failed” (unfortunately, the passenger with me at the time, a non-neuroscientist, did not appreciate my brain joke, rolled their eyes and called me “a nerd”).

Inhibitory control, which will be the focus of this review, has largely been defined as the withholding or prevention of inappropriate actions, often which stems from the conflict that arises when two discrete motor plans are activated simultaneously. While there are differences in opinion regarding what constitutes “conflict,” the common ground in this debate centers itself around the importance of the brain and its ability to first detect when newly perceived sensory information contrasts with the intended motor action of the organism through some common mechanisms such as value, and second to respond rapidly [1,2,3,4]. The car example in the first few paragraphs serves two purposes. First it helps exemplify the possible danger associated with a failure to correctly exert inhibitory control. Failure to successfully navigate competing motor sequences, can lead to an incorrect or maladaptive motor command being issued, which in turn decreases the likelihood for reward in a laboratory setting, and increases the likelihood for embarrassment or injury in the real world. Second, the car example also helps illustrate that while—hopefully—common enough to relate to, the frequency with which we either commit, or witness, complete failures to exert inhibitory control is relatively low.

The reason, barring a major neurological or neuropsychiatric condition, that the incidence of these failures is low is partly because it is highly adaptive *not* to lose control, but also because we have lots of practice successfully engaging control processes. Back to the car example, think about how many times you, like the motorist in our example, have been in a similar situation and failed to reapply the brake, instead running the red light—hopefully, that number is fairly small. Either way, it is even more likely that you can remember far more instances where you have successfully navigated intersections with multiple signals and never felt the urge to release the brake until it was appropriate. In fact, the difficulty in observing and eliciting these types of errors is illustrated in the laboratory by numerous psychological tasks such as the Stroop Task, stop-signal task, Flanker Task, etc. that report relatively low error rates despite the best efforts of experimenters to make subjects fail.

This remarkable resiliency to error is attributed to an ever growing network of brain regions and behavioral strategies tasked with facilitating inhibitory control. In addition to the act of mitigating conflict between competing responses on a given trial (i.e., inhibiting one response in favor of another), several other behavioral strategies such as proactive (i.e., mechanisms that insure the motorist from above is more attentive to sensory cues and more aware of how much pressure they are applying to the brake at the next red light) and reactive (i.e., mechanisms that caused the motorist from above to panic and accelerate quickly out of harm’s way) forms of control are also in place to help lessen/prevent the potential damage a breakdown in control can cause. Importantly, proactive and reactive mechanisms both facilitate successful inhibitory control, and we will attempt to highlight these processes throughout our discussion of inhibitory control.

Understanding which brain regions are responsible for the implementation of these various aspects of control has been described as a Rorschach Test for modern cognitive and systems neuroscience [3]. This analogy bears a great deal of truth as a quick survey of the literature yields evidence for the anterior cingulate cortex (ACC), the midcingulate cortex, the medial prefrontal cortex (mPFC), and the orbitofrontal cortex (OFC) all being necessary for the detection of competing motor outputs and the implementation of inhibitory control. Beyond the frontal lobe, parts, or all, of the striatum, the amygdala, and the thalamus have also been implicated in the implementation of control. In truth, finding a brain region not involved, at least in some way, in inhibitory control is quite challenging. While truly understanding the neural mechanisms that underlie inhibitory control will likely require decades more of theorizing and experimentation, this review will attempt to summarize and make sense of a subset of this literature that has chosen to look at inhibitory control in the context of the stop-signal task.

We will focus on the details of the stop-signal task in the following section, but for now, the choice to focus on stop-signal tasks as a case study for understanding inhibitory control stems from a much of our recent work employing the use of the electrophysiological and systems neuroscience approaches within the context of the stop-signal task in rodents. We see this as an advantage to discussions about inhibitory control as it allows for comparison of physiological processes across both brain regions and species, as well as affording us the opportunity to ask what happens when these brain regions are disrupted, something more difficult to do in a controlled manner in humans. Moreover, by limiting our review to studies that have employed the stop-signal task and recorded from brain regions throughout the frontal lobe, striatum, and midbrain we are able to more seamlessly generate a framework for the implementation of inhibitory control. One caveat to this approach is that the contributions of sensory processing modalities to this process are complex and numerous, however, given that these processes are ultimately highly specific to the modality being tested, it is for that reason that they are beyond the scope of this review. Instead, we will focus on the detection of conflict, the available evidence supporting proactive and reactive forms of control, how these forms of control impact action selection, and how detection of conflict ultimately can modulate the expression of a motor plan. Our hope is that this review provides a framework for future theories and experiments attempting to better characterize and understand the neural mechanisms of inhibitory control.

## 2. Stop-Signal Task

Throughout the literature, there are a variety of different behavioral tasks designed to study inhibitory control (i.e., the Stop-Signal task, the Stroop Task, Go/No-Go task, the Flanker Task, the Simon Task, etc.). Despite nuances in design and implementation, generally speaking, all of these tasks place a largely automatic or reflexive response in competition with a less automatic or more effortful response. This competition inevitably results in conflict when subjects are asked, at some point during the testing, to refrain from making the reflexive response in favor or the less reflexive response. Typically, in these scenarios the percentage of correct trials on reflexive and less reflexive trials is measured, and decreased accuracy on less reflexive trials, is suggestive of a breakdown in inhibitory control processes. Furthermore, decreases in accuracy are almost always accompanied by longer response or reaction times as well, indicative of a need for greater thought or effort.

Although there are great number of similarities between these tasks, the ease with which they have been adapted for study in non-human populations is variable. The Stroop Task for instance, arguably one of the most famous inhibitory control tasks, is difficult to adapt to rodents or primates in its human form, as animal subjects lack the instinctual automaticity (and ability) for word reading and color naming [5,6]. This highlights one advantage of the stop-signal task, as training rodents or primates to make and withhold operant responses based on visual or auditory cues, rather than word reading or color naming, is quite tractable, and makes direct cross-species comparison possible [6]. Additionally, while word reading and color naming take advantage of common human abilities, these response domains are restricted to specific use cases. In many ways, at least in the minds of the authors, the stop-signal task with its reliance on light and auditory cues in place of these more specific responses, more closely resembles a greater variety of real-world situations [6]. For instance, most scenarios, like driving a car or waiting your turn in line at the grocery store, require us to make different responses based on a variety of auditory or visual stimuli.

The idea of using the stop-signal task to directly study mechanisms of inhibitory control comes, in part, from the theoretical work of Logan and Cowan, which posits that stop-signal performance is determined by a race between a reflexive and a less reflexive response [6,7,8]. The premise of the stop-signal task requires subjects, on a majority of trials, to respond quickly, usually with a lever or button press, to some sort of GO cue (i.e., a light, tone, etc.). Performance on GO trials is then compared to performance on STOP trials, trials in which the initial GO cue is presented, but is then followed by a STOP cue (typically a different colored light or sound) that instructs the subject to either refrain from responding or make an opposite response (i.e., if the GO response instructs the subject to press the left lever, the STOP cue would instruct the subject to press the right lever instead). Difficulty on the task arises from the asymmetry of the task structure. On a typical stop-signal task, the number of GO trials exceeds the number of STOP trials by a recommended minimum of 3:1 [6]. Across species, this asymmetry in the number of GO and STOP trials, respectively, results in the development of reflexive, automatic, responding to the first cue, thus making it more difficult to refrain from responding on STOP trials [6,7,8,9,10,11,12,13,14,15]. Moreover, with the addition of a stop-signal delay, an experimentally manipulated delay between the presentation of the GO cue and the presentation of the STOP cue, researchers can better titrate task difficulty to individual subjects, creating both a measure of individual difference as well as a means to ensure inhibitory control processes are maximally challenged [6,7,8]. Variants of this task can also take advantage manipulations that alter reaction times (i.e., time to orient towards a cue) and movement times (i.e., time to make a response once a cue has been presented) distributions, respectively, in order to identify differences in types of inhibitory control, including proactive and reactive mechanisms [16,17,18,19].

The utility and adaptability of the stop-signal task has made it useful for everything from neurophysiological dissections of relevant brain circuitry [9,10,11,12,13,14,15,20,21,22,23,24,25,26,27,28,29,30,31], to assessment of inhibitory control across the lifespan [32,33,34], as well as between individuals both with and without neurological or neuropsychiatric conditions [17,18,19,35,36,37,38,39]. It is for these reasons that we chose to focus our review on the stop-signal task, as the stop-signal task, and its growing literature, allows us to study a rigorous behavioral task with validity in non-human and human settings, in combination with electrophysiological recordings that allow for the functional dissection of the brain regions responsible for inhibitory control.

## 3. Functional Dissection of Inhibitory Control

The stop-signal task, and its derivatives, require subjects to make a habitual response (i.e., respond with a left or right lever press to a directional light cue) on a majority number of trials (i.e., GO trials). This habitual response is then put in conflict on STOP trials, when the first directional cue is quickly followed by a second cue (i.e., STOP cue) telling the subject to either refrain from making a response or to redirect their response in the opposite manner. This behavioral sequence, requires the brain to represent two competing action plans, and is theorized to result in a race between action plans, where the winning plan corresponds to the response the subject makes.

**Figure 1:**
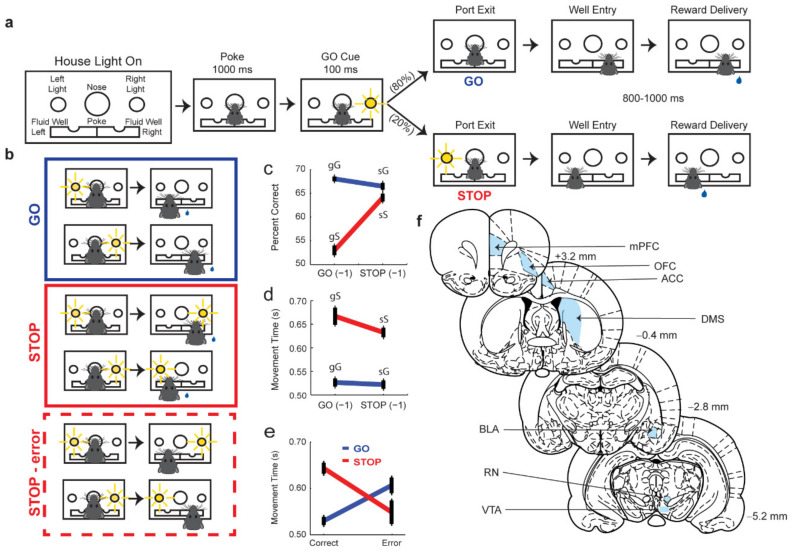
Stop-change task design. (**a**) Schematic of stop-change task. Following the house lights rats made a nose poke for 1000 ms before a light cue was illuminated on either the right or left side. On 80% of trials (GO trials) this light corresponded to the correct direction that the rat needed to move in order to receive reward. On 20% of trials a second light was illuminated after the initial GO cue directing the rat to inhibit their initial response to the first cue in favor of making a response in the direction of the second cue. (**b**) Illustration of GO (Blue), STOP (red), STOP-error (dashed red) trial types. (**c**,**d**) Percent correct and movement times for sequence effects. Sequence effects are reflected by the current trial type being capitalized (i.e., ‘G’ for GO; ‘S’ for STOP), and the trial type the came before the current trial type being lower case (i.e., ‘g’ for GO; ‘s’ for STOP). gG would be read as a GO trial that preceded by a GO trial. The four possible trial type are: gG = go, go; sG = stop, go; sG = stop, go; sS = stop, stop. Percent correct and movement times were averaged over sessions. Error bars represent ±SEM. (**e**) Movement times on correct and error GO and STOP trials. (**f**) Schematic of major brain regions discussed in this review (mPFC = medial prefrontal cortex; OFC = orbitofrontal cortex; ACC = anterior cingulate cortex; DMS = dorsomedial striatum; BLA = basolateral amygdala; RN = red nucleus; VTA = ventral tegmental area). Measurements relative to bregma. Figure 1c–e were adapted from Brockett et al., 2020 [15].

In order for competition resulting from two simultaneously active action plans to be mitigated, the brain must first represent each action plan, and, on correctly performed STOP trials, must either bias or possess the ability to suspend the first action plan in favor the correct motor sequence. In the following sections we will see that the dorsomedial striatum (DMS) represents dueling action plans, and that areas in the frontal lobe, such as ACC, mPFC, and OFC as well as areas such as the amygdala and ventral tegmental area (VTA) work in concert to exert influence over which action plan is seen to fruition. Finally, we will discuss new work suggesting that distinct motor pathways in the brain stem further augment responding on correct STOP trials (see Figure 1f).

### 3.1. Dorsomedial Striatum (DMS)

Pharmacological lesions and manipulations of DMS first implicated this area in inhibitory control [24,27,40,41]. Using a variant of the stop-signal task (see Figure 1), rats with DMS lesions were shown to perform worse on STOP-trials, requiring earlier warning in order to successfully inhibit the inappropriate response [27]. However, DMS lesioned rats also exhibited slower reaction times on GO trials as well, making it difficult to gauge the exact role of DMS in this process [27]. Similar impairments were observed in rats performing a 5-choice serial reaction time task, in which DMS lesioned rats were unable to successfully refrain from making a response during the delay period [24,40,41]. These findings were suggestive of the fact that DMS dysfunction corresponds to difficulty in the implementation of inhibitory control, however, the nature of this difficulty remained unclear.

Using a single unit electrophysiological approach, we recorded from the DMS of rats performing a novel variant of the stop-signal task in order to elucidate the exact role of DMS in this process. In our task (i.e., the stop-change task), a house light cues rats to the beginning of a trial, at which point rats must make and hold a nose poke into a central port for 1 s before a directional light cue, on either the left or right side of the port, is flashed for 100 ms. On 80% of the trials (i.e., GO trials), the directional cue instructs rats to move and hold in one of two fluid wells, beneath the center port, in order to receive a small liquid sucrose reward (i.e., a right directional cue, instructs the rats to move to the right fluid well). On the remaining 20% of trials (i.e., STOP trials), the initial GO cue is flashed for 100 ms, but is then followed by the presentation of the STOP cue, which instructs rats to cancel their initial movement in the direction of the GO cue, in favor of movement in the direction of the STOP cue (see Figure 1a for reference). Unlike the standard stop-signal task where both the correct engagement of inhibitory control processes or inattentiveness can result in correct responding, by forcing rats to make a STOP response, the stop-change task we have developed allows us to discriminate between correct engagement of inhibitory control processes and inattentiveness. Figure 1b illustrates the trial types typically analyzed in this task.

Using this paradigm, we have successfully shown that rats perform more accurately on GO trials (~75–80% correct) compared to STOP trials (~50–60% correct) and that their movement times (i.e., the elapsed time from leaving the center port to entering the fluid well) are predictive of performance (i.e., relatively fast, ballistic movement times on GO and STOP error trials, and slower more deliberative movement times on correct STOP trials) (Figure 1c–e) [9,10,11,12,13,14,15]. Moreover, on correct STOP trials, slower movement times showed a significant positive correlation with accuracy (Figure 1e) [9,10,11,12,13,14,15]. Error trials are defined as only trials where the rat makes the incorrect response.

Below we examine activity across multiple brain regions as rats performed this ask. When describing neural activity, we will describe ‘directional signals’ on GO trials and how they are altered during STOP trials. Since some neurons have response fields to the left whereas other neurons have response fields to the right we will refer to activity during movements to the left or right in terms of actions made into the response field and away from the response field based on the strength of firing. Actions that generate the strongest firing—averaged across both correct STOP and GO trials—will be referred to as ‘into the response field’ or the cell’s ‘preferred’ direction (i.e., thick lines in plots), whereas the opposite movement will be referred to as ‘away from the respond field’ or the cell’s ‘nonpreferred’ direction (i.e., thin lines in plots). The strength of the ‘directional signal’ is simply the difference between movements in opposite directions (i.e., how strongly cell firing discriminated between the two directions; difference between thick and thin lines in plots).

**Figure 2:**
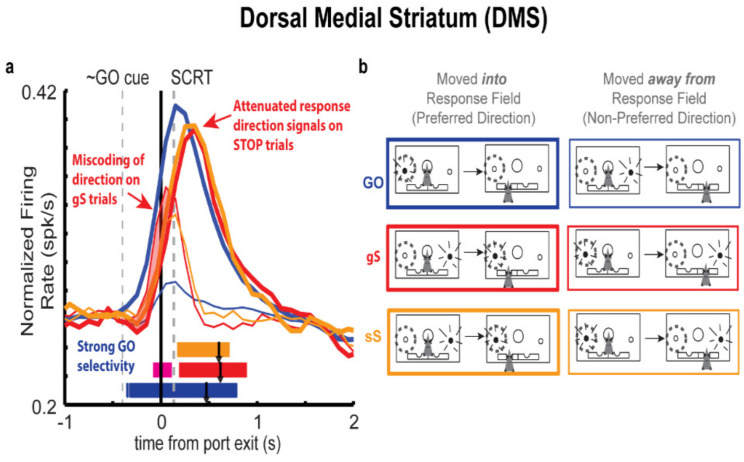
Miscoding of direction in the DMS. (**a**) Population histogram reflecting the initial miscoding of direction on GO (blue), gS (red), and sS (orange) trials. Strongest GO selectivity is observed on GO trials (blue bar), followed by gS (red bar) and sS (orange bar). Colored bars represent significant differences in directional selectively as computed by sliding t-test comparing preferred (thick) and non-preferred (thin) direction over 100 ms bins for each respective trial type (p’s < 0.01). Grey dashed line labeled GO cue represents the approximate average presentation time of the GO cue. Grey dashed line labeled SCRT represents the stop-change reaction time. Please note that GO trial refers to all GO trials, irrespective of the trial type that came before. (**b**) Schematic depicting response profiles representing movements into (thick lines) and away from (thin lines) a neuron’s response field (gray dashed circle). Figure adapted from Bryden et al., 2012 [9].

Electrophysiological dissection of DMS neural responses of 437 neurons revealed strong directional preferences on both GO and STOP trials for increasing-type cells (i.e., cells that exhibit increased firing during the response epoch) (Figure 2a,b) [9]. As reflected by the thick blue line in Figure 2a, on GO trials, neurons encoding movements into a neuron’s response field (i.e., the preferred direction of the neuron; quantified as a selective increase in firing for either a left or right movement) showed rapid increases in firing rate emerging just prior to port exit, reaching a peak approximately 0.2 s after port exit [9]. Conversely, neurons encoding movements away from a neuron’s response field (i.e., the non-preferred direction; Figure 2a; thin blue line) revealed minimal increases in firing rate during this period. Firing rate was correlated with movement times, suggestive of the fact that this activity is directly related to the directional response of the rat [9].

Analysis of DMS firing patterns on STOP trials (Figure 2a) revealed an initial miscoding of direction that, on correctly performed STOP trials, was rectified prior to our calculation of the stop-change reaction time (SCRT), a behavioral measure of time required to stop and then redirect a behavioral response (i.e., calculated as the difference in movement times on correctly performed STOP trials and GO trials) [9]. On correctly performed STOP trials, an initial increase in firing from the population of neurons encoding the non-preferred direction was observed just prior to port exit on a time course similar to that seen on GO trials (Figure 2a; thin red line) [9]. Presumably this firing represents the action plan in response to the initial GO cue that must be overwritten. Notably, this initial encoding of the first cue (i.e., GO cue) was stronger on gS trials (i.e., STOP trials that were more difficult to inhibit because they were preceded by a GO trials; red) compared to sS trials (i.e., STOP trials that were easier to inhibit because the preceding trial was also a STOP trial; orange) consistent with increased errors and slower movement times on gS trials. Importantly, for both gS and sS trials, population activity just after port exit, following the presentation of the STOP cue, quickly signaled the appropriate direction (i.e., the direction now signaled by the STOP cue). This second wave of firing emerged around the time of port exit and at the time the SCRT (Figure 2a, thick and thin red lines diverge for both red and orange). This adaptive shift in the firing pattern of the population of DMS neurons predicted successful STOP trial performance as well as slower movement times [9].

Interestingly, on STOP-error trials (i.e., trials where rats responded in the incorrect direction) firing favored the incorrect direction, and population-level response profiles were not statistically discriminable from that of GO responses [9]. Firing in the preferred direction emerged rapidly on the time course and at magnitude comparable to that which was observed on GO trials. Critically, this increase in population level responding correlated with faster movement times and subsequently decreased accuracy. At the population level, firing encoding the non-preferred direction did begin to emerge on STOP-error trials, unlike on GO trials, however, this response emerged just after the SCRT [9].

Collectively, these findings suggest that the DMS plays a direct role in representing alternative action plans [9]. Analysis of single unit data on STOP trials revealed clear representation of both directional responses and correct performance on these trials, correlated with the rapid overtaking of the population of cells encoding the non-preferred directional response, by the population of cells encoding the preferred directional response. Importantly, this change in directional encoding occurred before the SCRT, a behavioral estimate of the time it should take an animal to stop and redirect its behavioral response. Moreover, analysis of error trials revealed errant encoding of the preferred direction, suggesting that on these trials, rats were unable to inhibit the maladaptive response. Although DMS appears to be one area in which the race between correct and incorrect action plans is occurring, and suggestive of the fact that disruption of DMS may result in errant responding, it remains unclear what factors facilitate the inhibition of the incorrect response, and the biasing of behavior in the correct direction.

### 3.2. Anterior Cingulate Cortex (ACC)

Numerous behavioral results/theories have implicated the ACC in having a central role in the implementation of inhibitory control [1,2,42,43,44,45,46,47,48,49,50,51]. These theories have evolved from early speculation that emotive processes radiated from ACC in an effort to bias behavior accordingly [43] to theories that instead suggested a role for ACC in the motivational biasing of behavior [44]. In the late 1980’s/early 1990’s theories of ACC function employed a more cognitive psychology lens, suggesting a that ACC biased behavior through attentional mechanisms [45,46], conflict detection [47,48,49], and most recently through a kind of value assessment about whether to stay or forage [1,2]. In all instances, ACC has largely been depicted as a center for the integration of emotionally valenced and value based information that in turn biases decisions regarding two competing outputs (i.e., stop or go, stay or forage, etc.).

While highly debated, arguably the most influential account of ACC function is the “conflict-monitoring hypothesis” [47], which suggests that one role of ACC is in the detection of instances in which a subjects intended action is at odds with the action dictated by the subject’s environment. Importantly, while the authors never intended for this to be an all-encompassing account of ACC function [47,48,49], using this computational approach, the authors were able to account for a variety of previous ACC-related findings including its role in response override [5,52,53,54,55,56,57,58,59,60], undetermined responding [61,62,63,64,65,66,67,68,69,70,71,72,73,74] and tasks involving error commission [75,76,77,78,79,80,81]. Importantly while this framework highlights a role for ACC in both representing the goal as well as possessing the ability to bias action selection toward the appropriate, this biasing, necessarily requires inhibitory control, and for that reason we will discuss the involvement of ACC in inhibitory control from that perspective.

The framework of the conflict-monitoring hypothesis, much like Logan and Cowan’s framework for stop-signal performance [7], conceptualized performance on the Stroop Task as a race between opposing action plans (i.e., wording reading vs. color naming), where, upon realizing two action plans were simultaneously active, ACC would alert in an effort bias attention and direct behavior accordingly [47]. Recent accounts of ACC function have subsumed this original proposal for ACC function and reframed the role of ACC as one that is assessing the costs and benefits of breaking from a more automatic holding pattern and entering into a more exploratory pattern [1], but even still at its core all accounts attempt to explain what ACC is signaling when competing action sequences are activated simultaneously.

It is for these reasons, that we sought to investigate the physiological profile of ACC during the performance of our stop-change task. As described previously, rats were trained on the stop-change task, fitted with a drivable 8-channel electrode targeting ACC, and tested on the stop-change task [12]. As before, rats exhibited greater accuracy on GO trials relative to STOP trials, as well as shorter movement times on GO versus STOP trials. Moreover, when GO and STOP trials were split between correct and incorrect responses, movement times on STOP error trials were extremely fast relative to correctly performed GO and STOP trials, suggesting that rats were not waiting to choose a response, but acting reflexively.

**Figure 3:**
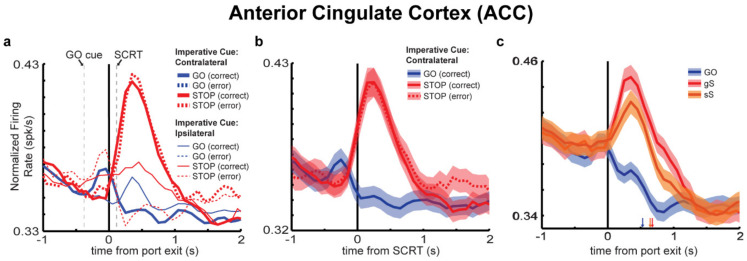
Enhanced firing of ACC neurons on STOP trials. (**a**) Population histogram depicting average firing on GO (blue), GO error (dashed blue), STOP (red), and STOP error (dashed red) trials. Thick and thin lines representing firing into (thick) and away from (thin) a neuron’s response field. Grey dashed line labeled SCRT represents the stop-change reaction time. Importantly, the number of single units showing increased firing for STOP versus GO trials significantly outnumbered those showing increased firing on GO versus STOP trials both pre-SCRT (34 vs. 8, binomial test, *p* < 0.05) and post-SCRT (134 vs. 31, binomial test, *p* < 0.05) [12]. (**b**) Population histogram from the same neurons as in A, however, only GO correct (blue), STOP correct (red) and STOP error (red dashed) firing rates cued to movements into a neurons response field are shown. Activity is aligned to the SCRT. Ribbons represent standard error of the mean. (**c**) Response of ACC neurons to degrees of conflict induced by the previous trial. GO (blue; little conflict), gS (red; high conflict), and sS (orange; medium conflict) for movements into a neurons response field are shown. Figure adapted from Bryden et al., 2019 [12].

We analyzed neural activity from 536 ACC neurons and found that at both the population and single unit levels, ACC neurons exhibited a strong preference for STOP trials (Figure 3a–c). Specifically, ACC neurons fired strongly on STOP trials when rats correctly refrained from responding in the non-preferred direction in favor of firing in the preferred direction (i.e., the direction signaled by the STOP cue) (Figure 3a,b). As seen in Figure 3, GO trials elicited limited firing from ACC neurons and did not appear to be particularly selective. Furthermore, greater firing on STOP trials was correlated with greater accuracy and slower movement times on correctly performed STOP trials, but not GO trials, further suggesting that firing in ACC is unique to STOP trial performance.

We also analyzed whether the magnitude of firing rate was impacted by the degree of conflict (Figure 3c). One advantage of the stop-change task is that trial history can be used as proxy for predicted difficulty on the current trial. For instance, a string of STOP trials might prepare a rat to proactively exercise inhibitory control on the current trial, resulting in slower movement times overall and better accuracy, whereas a string of GO trials prior to the current STOP trial may promote speeded responding, and make it more likely that a rat commits an error when presented with a STOP trial. As predicted, gS trials (Figure 3c; red) (i.e., trials where a GO trial preceded the current STOP trial) elicited the highest firing in ACC when compared to sS trials (Figure 3c; orange) (trials where a STOP trial preceded the current STOP trial) and GO trials (Figure 3c; blue), suggesting that firing in ACC at both the population and single unit level, is significantly modulated by previous instances of conflict.

**Figure 4:**
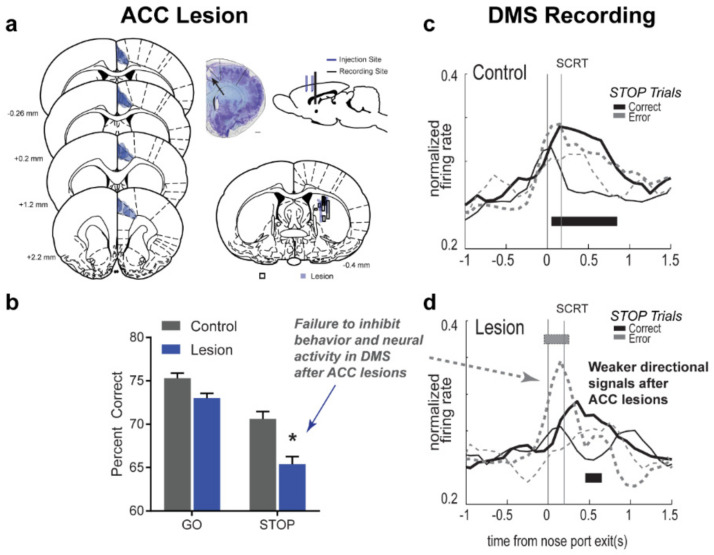
**Unilateral ACC lesions impair response selection.** (**a**) *Left*: stereotaxic overlays detailing the extent of the lesion for all 8 rats (4F, 4M) tested. Lesions were primarily constrained to rodent Cg1 and showed little overlap with other frontal regions [83]. Unilateral lesions were performed to minimize the impact on the animal in order to assess changes in DMS firing without excessive changes to behavior or potential engagement of redundant systems [13,84]. *Middle*: Photomicrograph of Nissl stained tissue showing representative lesion (arrow). Overlay approximately matched to section (~ +1.6 mm anterior from bregma). Scale bar = 1 mm. *Top Right*: Sagittal view of two injection sites in ACC and the targeted recording site in DMS. *Bottom Right*: Overlays detailing the tracks of all 16 electrodes tracks in DMS. Shaded blue tracks signify lesioned animals, while black borders signify controls. Injections and electrodes were placed in either the right or left hemisphere, and placement was counterbalanced across treatment groups. Note: placements are shown on the same hemisphere to reflect the unilateral nature of the manipulations. (**b**) Percent correct scores on GO and STOP trials by condition averaged over recording sessions. (**c**,**d**) Population histograms for control ((**c**); *n* = 97) and lesion ((**d**); *n* = 53) rats aligned to onset of the second cue light. Solid lines represent firing on STOP-change trials where the rat made the correct response, and dashed lines represent STOP-change trials where the rat committed an error. Thick and thin lines represent firing into (thick) or away from (thin) a neuron’s preferred direction. Tick marks (grey) represent significance when comparing thick to thin lines within trial type. Figure adapted from Brockett et al., 2020 [13].

Collectively, these findings largely confirm the conflict-monitoring hypothesis in suggesting that ACC is uniquely activated by the experience of conflict (i.e., when two competing motor sequences are active simultaneously), or said another way, the need to exercise inhibitory control. Moreover, these findings are largely supported by recent imaging data in patients with Parkinson’s disease, that showed reduced bilateral functional recruitment of ACC in patients performing a Go-NoGo task [82]. However, based on these findings, whether firing in ACC modulates downstream action selection was unclear. To remedy this, we trained a different group of rats on the stop-change task, and performed unilateral lesions of ACC while recording downstream from DMS (Figure 4a). In this study, we predicted that if ACC firing represented a behaviorally meaningful signal, that this would be reflected in DMS, which receives monosynaptic projections from ACC, and as described in the section above, represents competing action plans. Specifically, we hypothesized that ACC lesioned rats would perform worse on STOP trials, and that action selectivity in DMS would be altered on STOP-error trials.

Rats were trained on the stop-change task, prior to conducting pharmacological lesions of ACC and electrode implantation in DMS [13]. Following surgery, ACC lesioned rats performed worse on STOP trials relative to controls (Figure 4b). Moreover, ACC lesioned rats were worse on high conflict (i.e., gS) and lower conflict (i.e., sS) trials compared to controls. Interestingly, ACC lesioned rats still performed better on sS trials and gS trials suggesting that while a fundamental impairment in the regulation of inhibitory control exists, unilateral damage to ACC ultimately leaves the ability to react to the experience of conflict (i.e., conflict adaptation) intact. Analysis of firing rates of DMS neurons in controls revealed characteristic representation of response direction, where on correctly performed STOP trials, firing in the non-preferred direction was quickly overtaken by firing corresponding the preferred direction prior to the SCRT (Figure 4c; black lines). However, in lesioned rats, this change in directional preference was delayed, with peak firing from neurons representing the non-preferred direction emerging well after the SCRT (Figure 4d; black lines), suggesting that the animal’s ability to react to conflict may be impaired. While still able to perform STOP trials correctly, the mechanisms of action selection were delayed in ACC lesioned animals suggesting that ACC plays a role in the biasing of action selection in the DMS. Analysis of single units in the DMS revealed a similar pattern with significant directional encoding emerging in separate analysis epochs related to the first (i.e., GO) and second (i.e., STOP) cues in controls rats, but not in ACC lesioned rats, which did not reach a significant threshold during the analysis window corresponding to the STOP cue. Additionally, when looking at population firing rates during the second cue epoch, on STOP error trials, no significant difference in firing rates encoding the non-preferred and preferred directions emerged in controls (Figure 4c; gray dashed), but strong directional encoding for the preferred, albeit incorrect, direction emerged in ACC lesioned rats (Figure 4d; gray dashed). In ACC lesioned rats, the strength of the firing on STOP-error trials was similar in magnitude and timing to firing observed on GO trials, and is suggestive of failure to react to conflict between competing inputs, similar to the motorist in the introduction who drove into the middle intersection in response to a green turn arrow.

Despite not observing a behavioral impairment in conflict adaptation in ACC lesioned animals, we asked whether there were clues in signals associated with differing degrees of conflict that could point toward a mechanism by which ACC might bias firing in DMS. By comparing firing rates on gS and sS trials, we found that in controls, directional selectivity emerged earlier on gS trials relative to sS trials and persisted longer, indicative of a greater need for control. The extended period of directional selectivity to the first cue on gS trials suggests that conflict-induced activation of ACC may be important for shutting down representation of the first cue/more automatic response in control rats in order to facilitate reactive inhibition. Remarkably, this pattern was not observed in lesioned rats, where directionally selective firing was observed equally and with similar timing for gS and sS trials. This observation was recently support by single unit work in humans [21]. Analysis employing targeted dimensionality reduction revealed that in humans performing the stop-signal task, ACC activity on STOP trials helped amplify task-relevant information during conflict, suggesting that ACC biasing action selection by increasing the awareness to the STOP cue [21].

While the conflict-monitoring hypothesis was never designed with the intention to fully account for all of ACC’s function, it brought to light the possibility that ACC may be uniquely sensitive to competition between two opposing motor outputs/the need for inhibitory control. Although this framework has since been revised and restructured several times, current models of ACC function still suggest a role in amplifying task relevant information in the service of biasing action selection. These single unit findings support this notion, and provide a concrete link between the detection of conflict in ACC and biasing of behavior downstream. Interestingly, the results of the ACC lesion study in rats suggest that other mechanisms of inhibitory control, such a proactive control or conflict adaptation remain relatively intact [13]. While this could be due in part to the unilateral nature of our manipulation, other single unit data exploring the OFC in the context of the stop-signal task suggests that this function may be served by a different area entirely.

### 3.3. Orbitofrontal Cortex (OFC)

Historically, the OFC has long been implicated in inhibitory control. Studies examining patients that underwent frontal lobectomies and OFC leucotomies frequently reported symptoms of behavioral disinhibition, in which patients exhibited intense shifts in mood/personality [85,86,87,88,89,90,91]. More recent work has demonstrated in the OFC grey matter volume is inversely correlated with the time needed to stop or cancel an initiating action, further suggesting a role for OFC in these processes [92]. Collectively, these findings in human patients were largely supported by a growing body of work employing a variety of animal models that suggested OFC was critical for reversal learning, a task where animals are first reward for correctly using one strategy (e.g., pushing the right lever to receive reward), and then are switched to being rewarded for using another strategy (e.g., pushing the left lever to now receive reward) within the same recording session [93,94,95,96,97,98,99,100,101,102,103,104,105,106]. Unfortunately, this initial theoretical insight, failed to account for other observations, such as the ability of OFC lesioned subjects to learn an initial strategy, and later the observation that when shifted between three strategies, OFC lesions seemed to leave behavioral performance unhindered [91,104,105,106,107]. For a variety of different experimentally and theoretically grounded reasons, the view that OFC was important for inhibitory control was gradually overturned in favor of theories that placed a greater emphasis on OFC’s role in outcome expectancy and more recently in representing a kind of task space (for a recent review of OFC function see [108]).

Despite evidence to the contrary, OFC repeatedly has been shown to be important for stop-signal performance, in a way that consistently highlights a role for OFC in inhibitory control [108]. OFC lesions disrupt performance on the five-choice serial reaction time task, a inhibitory control task commonly used in rodents [94]. Lesioned rats commit more perseverative errors, suggesting an inability to integrate newly acquired information about the probability of an action’s ability to garner reward. Moreover, using the stop-signal task, OFC lesions in rats have produced longer stop-signal reaction times (SSRTs), a behavioral measure, similar to the SCRT, that serves as an estimate of how long it takes to cancel an action [109]. Similarly, infusion of the attention-deficit hyperactivity (ADHD) drug, atomoxetine, into OFC has similarly shown to stabilize behavioral performance and improve SSRT measures [110]. These findings suggest that OFC seems to regulate a subject’s ability to respond to instances of conflict, and may suggest a role for OFC in adapting behavioral strategies following the experience of conflict.

The ability to adapt behavioral responses following the experience of conflict is often termed proactive control or conflict adaptation. Importantly, while this mechanism of control may not prevent an initial error in action selection from occurring, this mechanism helps ensure that, behavioral responses are proactively slowed down so as to increase the probability of correctly engaging mechanisms of inhibitory control in the future. Accordingly, in a recent paper, non-human primates were trained to perform the stop-signal task [20]. OFC recordings revealed that neurons in OFC encoded successful task completion, and distinguished between successful versus failed STOP trials [20]. Moreover, researchers showed that inhibitory signals detected in the firing pattern of OFC neurons were orthogonal to value selective signals detected in those same neurons, suggesting that while multiplexed, at the level of single cells in the OFC, inhibitory control signals are distinct from value signals [20].

**Figure 5:**
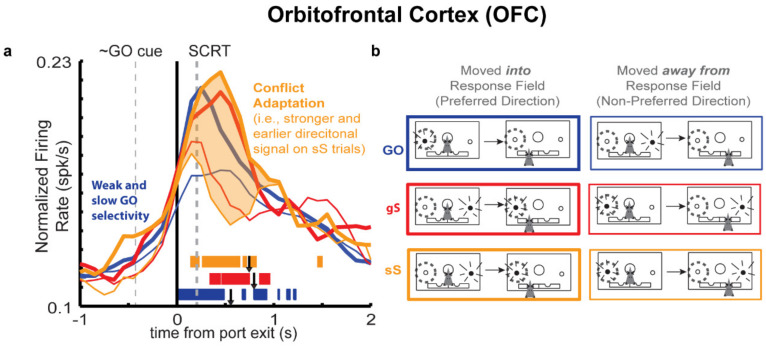
Strength of the directional signals are modulated by previous trial conflict in OFC. (**a**) Population histogram for all increasing-type OFC neurons (*n* = 209) on GO (blue; little conflict), gS (red; high conflict), and sS (orange; medium conflict) trials. Conflict adaptation is exhibited in the form of stronger and earlier directional signaling on sS (orange bar/shaded region) versus gS (red bar) trials. (**b**) Schematic depicting response profiles representing movements into (thick lines) and away from (thin lines) a neuron’s response field. Figure adapted from Bryden and Roesch, 2015 [10].

These findings largely support findings from our work investigating OFC signals during the stop-change task. Once again, rats were trained on the same stop-change task and exhibited similar behavioral patterns on both GO and STOP trials as described earlier [10]. Analysis of firing data from OFC neurons revealed that neurons in OFC were modulated by both direction and movement speed, and to a lesser degree by trial type, firing significantly for both GO and STOP trials alike. The strength of firing on STOP trials was highest on trials where conflict adaptation was observed (i.e., sS trials) (Figure 5a,b). As illustrated by the orange shaded region of Figure 5a, firing on sS trials peaked above that observed on GO and gS trials. Moreover, analysis of the timing (Figure 5a; orange, red, and blue bars reflect significant differences between thick and thin lines within overlapping 100 ms time bins) illustrate that relative to gS trials (red), selective firing on sS trials (i.e., the difference between thick and thin orange line) emerged earlier and was associated with greater accuracy. On sS trials, the experience of conflict on the previous trial, appeared to elicit a unique response in OFC neurons, as observed by a negative correlation between firing and percent correct. These observations suggest that when rats struggled to inhibit the automatic response on the previous trial, OFC provided a boost to the directional signals that was needed to bias action selection. This interpretation is broadly consistent with recent work in monkeys suggesting that OFC is involved in reconciling cognitive signals during conflict adaptation [111].

While future work is necessary to elucidate the exact downstream consequences of this proactive OFC signal, behavioral and neurophysiological evidence seems to suggest that OFC facilitates proactive control, by slowing behavior and increasing the odds of correctly responding during a STOP trial that preceded by another STOP trial. In principle this might explain why despite disruption of ACC function, and impaired overall performance on STOP trials, ACC lesioned rats still exhibit intact conflict adaptation signaling. While ACC and OFC likely work in concert, the results from these two studies suggests distinct roles for ACC and OFC in the regulation of inhibitory control.

### 3.4. Medial Prefrontal Cortex (mPFC)

As with the ACC and OFC, the mPFC has long been implicated in flexible behavior and inhibitory control as well. In rats, lesions to rat prelimbic cortex result in SSRT deficits on the stop signal task [110]. Additionally, using a task a cognitive/behavioral flexibility, the attentional set-shifting task, pharmacological ablation of neurons [112] and astrocytes [113] result in impairments on the extradimensional shift portion to the task. During the extradimensional shift, rats are rewarded for using a previously unrewarded exemplar to guide decision-making. Although cognitive flexibility refers to an organisms’ ability to switch between stimuli dimensions or strategies in order to respond adaptively, this phenomenon necessitates the ability to inhibit a more automatic response in favor of a less automatic response, and this how often made it difficult to parse differences in the neurobiological mechanisms underlying flexibility and those underlying inhibitory control [114].

In order to understand the contributions of mPFC to stop-signal performance, we recorded from the mPFC of rats performing our stop-change task [11]. Behavioral performance was identical to those described in the previous sections. Analysis of firing rates of mPFC neurons suggested that mPFC was responsive to both GO and STOP trials. For both trial types, rapid increases in firing for the preferred direction emerged following port exit. The timing of this increase was similar regardless of trial type, however, peak amplitudes were reached between (~0.5–0.8 s) after port exit, slower than what is typically seen in DMS, ACC, and OFC. Population and single unit firing was also modulated by degree of conflict, with highest firing rates being observed for STOP trials that followed a GO trial (gS), followed by STOP trials that preceded a STOP trial (sS). This pattern of modulation is similar to what was observed in ACC, albeit the timing of the response pattern in mPFC was delayed. This delay in conflict modulated firing suggests a different role for mPFC in stop-signal performance than that of ACC. Whereas ACC has been demonstrated to fire rapidly on STOP trials, and important for the modulation of action selection signals in DMS, the firing rates observed in mPFC suggest a role in updating, perhaps on the effectiveness of the employed strategy, which is likely to benefit future performance. Future work would benefit from investigating the effects of mPFC impairment on DMS action selection.

### 3.5. Summary of Frontal Function

Work investigating the contributions of frontal cortex to inhibitory control has started to reveal functionally distinct roles for ACC, OFC, and mPFC, respectively. While ACC appears to have a clear role in signaling and reacting to competition between simultaneously active actions, both OFC and mPFC, although sensitive to instances of conflict, appear to focus their efforts proactively adapting behavior for success on future trials. OFC in particular is responsive to errors, and appears to slow behavior, a form of conflict adaptation, to help ensure improved performance should competition arise again. On the other hand, mPFC, with its delayed conflict modulated firing, appears to be essential for taking in information on strategy effectiveness, although future work is necessary to clarify how mPFC is doing this.

Importantly, although frontal regions appear to play an important role in the biasing of action selection, it is important to consider that these brain regions do not act independently from the rest of the brain and information from other brain areas such as amygdala, ventral tegmental area (VTA), as well as motor areas such as red nucleus (RN) may also play an important role in stop-signal performance.

### 3.6. Amygdala

The amygdala is small almond shaped brain region that is comprised of a collection of interconnected nuclei and is situated deep within the temporal lobe [115,116,117]. Although historically the amygdala has been investigated for its role in fear and emotion, a more modern view of amygdala function is one that incorporates the amygdala’s involvement in associative learning more generally, and in insuring that valenced information is brought to the attention of frontal brain areas tasked with decision-making and the biasing of action selection [115,116,117].

Anatomically, evidence for the importance of amygdala, specifically, the basolateral amygdala (BLA), in influencing frontal function comes from the presence of strong reciprocal connections between BLA and ACC, OFC, and mPFC [115,116,117]. Disruption studies of BLA function in the context of reversal learning paradigms have shown impairments in reversal learning [105,118], but also that the loss of associative encoding in BLA leads to weakened signaling in OFC, and failure to accurately map value onto actions [118].

Evidence of anatomical interconnectedness and the importance of BLA in detecting changes in reward contingencies led us to speculate that BLA may be important for detecting the need for inhibitory control and possibly mitigating conflict that arises from competing action plans [119]. Using a discrimination task, a precursor to our stop-change task, rats were trained to discriminate between three distinct odors [119]. Two of the odors were associated with a forced choice action with the third odor was associated with free choice. Responses on forced choice trials were identified as either congruent or incongruent. On congruent trials, rats were directed to make a response in the direction rats were biased toward during free-choice trials, while on incongruent trials rats were forced to make a response in the opposite direction. Rats responded faster on congruent trials, when the odor directed them to make a response in their preferred direction. Single unit recordings from BLA showed that neurons fired preferentially only on incongruent trials when an error was about to be committed. On these error trials, firing emerged early, before response initiation and was correlated with error commission and a lack of reward. This preemptive signal may be a type of teaching signal that could be important for helping slow behavior on future trials. Given that the behavioral task used in this study was not identical to the stop-change task described previously, it is difficult to make comparisons about the timing of these results, but they are nevertheless suggestive of a role for amygdala in inhibitory control processes. Future work must examine BLA firing on the stop-change task in order to more fully dissect its contribution to action selection and conflict adaptation.

### 3.7. Ventral Tegmental Area (VTA)

In the laboratory, and in the real world, inhibiting inappropriate actions, and subsequently performing correct or adaptive ones, is typically associated with reward or positive outcomes. Going back to our car example from the beginning of the review, while it is hard to classify correctly refraining from entering the intersection errantly as rewarding per se, it is certainly adaptive and may be associated with a variety of positive outcomes. It is perhaps not surprising then that dopamine (DA), a neurotransmitter commonly associated with reward and motivation, has been shown to be important for stop-signal performance [14]. The VTA is comprised of a small cluster of neurons located in the midbrain and is the place of origin for DA neurons that are part of the mesocorticolimbic system. The VTA has been shown to be important for numerous cognitive and reward-related processes [120,121], but its exact role in inhibitory control has often been debated.

Pharmacological manipulation of the DA system in subjects performing the stop-signal task has resulted in mixed results [24,109,122,123,124,125,126]. Some studies report impaired responding on GO trials [24,109] while others report improved performance on STOP trials [122,123,124,125,126]. In order to better understand the role DA signals from VTA played in inhibitory control we recorded from a different set of rats performing our stop-change task [14]. Once again, we observed similar behavioral results as described previously. Neurons were screened on the basis of waveform characteristics to classify populations of putative DA cells. Populations of putative DA cells seemed to discriminate between GO and STOP trials, respectively. Relative to GO cues, putative DA neurons fired less for STOP cues, however, this same population of cells appeared to fire more upon receiving reward during STOP trials. The diminished firing observed following the presentation of a STOP cue, and exaggerated firing period upon receiving reward on correctly performed STOP trials is suggestive of increased uncertainty concerning the likelihood of reward on STOP trials. Firing was further modulated by trial history, with lower firing rates observed on STOP trials that were preceded by long strings of GO trials. In effect, firing rates on STOP trials and future accuracy appeared to follow a kind of dose-response curve, diminishing with each additional previously experienced GO trial. In other words, the more GO trials a rat experiences in a row prior to a STOP trial, the less likely it is to perform the STOP trial correctly, which reflective in diminished firing of putative DA neurons in VTA in response to the STOP cue. Collectively these results suggest that the firing of putative DA cells in VTA functions as a kind of gauge for the rat’s estimation of its probability of success on a given trial. Whether and how this information is used to influence frontal processes, inhibitory control, and subsequently action selection remains unclear, and an area for future research. Moreover, it is important to consider that the substantia nigra, a brain region located lateral to VTA, and that is another hub for dopaminergic neurons, has also been implicated in inhibitory control mechanisms and is altered in Parkinson’s disease patients showing deficits in inhibitory control [17,19,127]. Although the link(s) between reward certainty in VTA or substantia nigra, or both, error commission in amygdala, and action selection/inhibitory control remain speculative, it seems likely that these signals work in tandem to better calibrate and assist frontal cortex in appropriately implementing control.

### 3.8. Red Nucleus (RN)

Relative to more “cognitive” brain areas and brain areas important for reward, comparatively less attention is paid to non-cortical motor areas of the brain tasked with the actual planning and execution of motor responses. This inequity within the field is problematic as correct cognitive computations are often useless without accurate, well-timed, motor outputs. Understanding whether and how the motor system influences and responds to inhibitory control signals is crucial to our understanding of inhibitory control. In order to begin to address this, we recently recorded from the RN of rats performing the stop-signal task [15].

The RN is a key node in the descending motor pathway and receives projections from the interpositus and dentate nucleus of the cerebellum as well as from the premotor cortex [128,129,130,131,132]. At a cellular level, the RN is further subdivided into magnocellular and parvocellular divisions with projections from the interpositus nucleus targeting the magnocellular division and premotor and dentate nucleus projections targeting the parvocellular extant [129,130,131,132,133,134,135]. Whether these divisions are functionally meaningful and whether RN plays the same role in rodents as it does in primates is debated, as the ratio of magnocellular to parvocellular cells has fluctuated across evolution [130,131,136], however, collectively, the RN has been overwhelmingly implicated in goal-directed movement [137,138,139,140,141,142,143,144,145,146,147] and motor/gait adjustment [137,140,143,145,147,148,149]. Based on this evidence, we predicted that the RN in rats might be instrumental in performing rapid adjustment in behavior and possibly altering motor responses based on past experience.

**Figure 6:**
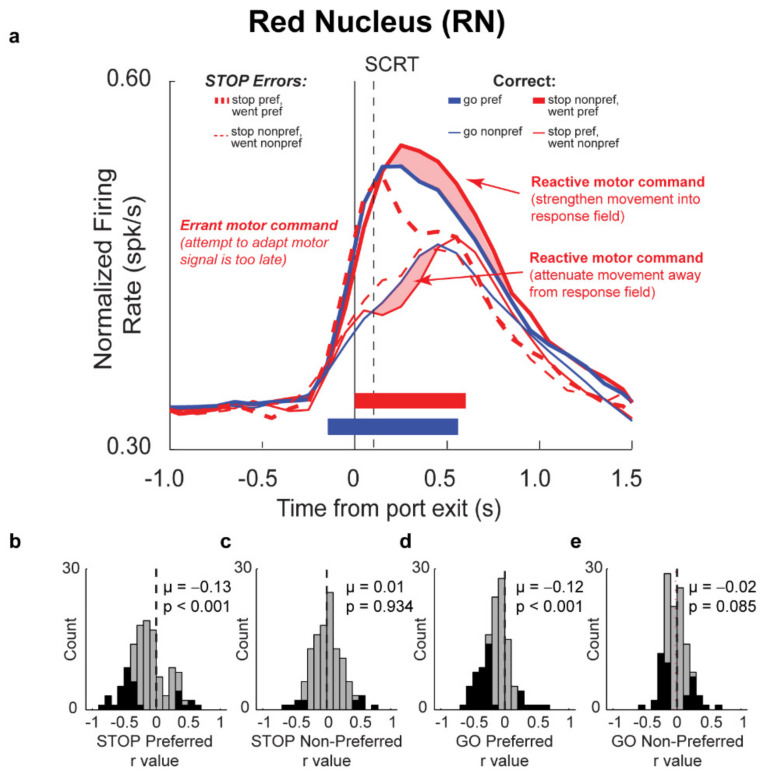
Neurons in RN reflect combined movement on STOP trials. (**a**) Average population histogram for increasing-type neurons (*n* = 121) for GO (blue), STOP (red), and STOP-error (red dashed) trials. Thick and thin lines represent movements into (thick) and away from (thin) a neuron’s response field. (**b**–**e**) Distribution of r-values depicting correlation between firing rate during the response epoch and movement time on STOP-preferred (**b**), STOP-non-preferred (**c**), GO-preferred (**d**), and GO-non-preferred (**e**) directions (Wilcoxon; μ = mean). Black bars indicated individual neurons that exhibited a significant within session correlations between firing rate and movement time (*p* < 0.05). Figure adapted from Brockett et al., 2020 [15].

Rats exhibited identical behavioral phenotypes as described previously performing better on GO relative to STOP trials. As shown in Figure 6a, analysis of single units in RN during task performance revealed directionally selective increases in firing on both GO and STOP trials. As with mPFC and VTA while the timing of increases in firing on both GO and STOP trials was similar emerging ~0.1–0.2 s before port exit, the trajectory of these changes in firing were different. Relative to GO trials, directional firing on correctly performed STOP trials was noticeably increased and broader for the preferred direction (Figure 6a; red shaded region). On STOP error trials firing in the preferred direction quickly decayed relative to both GO and correctly performed STOP trials shortly after port exit (Figure 6a).

In all cases, firing rates were strongly correlated with movement speed, suggesting that RN neurons may be responsible for driving movement. To determine whether increases in firing drove movement speed we performed a regression analysis to determine the numbers of neurons within the response epoch that correlated with movement time (Figure 6b–e). Counts of neurons on GO and STOP trials were significantly shifted in the negative direction only when the response was made in the preferred direction on both GO (Figure 6d) and STOP (Figure 6b) trials, but not for movements made in the non-preferred direction for either trial type (Figure 6c,e). The significant negative correlation between firing rate and movement time is suggested of a speeding or driving of responding by neurons in RN.

We reasoned that the amplification of firing that is specific to correct STOP trials that occurred after the SCRT and may be reflective of the initiation of the GO movement that is quickly modified by the presence of the STOP cue (Figure 6a; red shaded region). Given that movement times were strongly correlated with firing rate; this amplification may also suggest that RN is attempting to reactively boost or speed responding along in order make sure the motor response falls correctly in the response window. In order to verify that this boost in firing was behaviorally adaptive, we examined firing rates as a function of trial history. We found that on GO trials that were preceded by another GO trial (gG) that directional selectively emerged 100ms before GO trials that were preceded by a STOP trial (sG), suggesting that increasing-type cells in RN contribute to trial-by-trial adjustments in behavior via a mechanism of proactive adjustment, slowing future movements to the first cue following the experience of conflict.

Much like our work from frontal areas, we have shown that the RN, a nucleus that is typically one of the last areas within the brain to operate on motor signals before they are sent to the spinal cord, is also readily modulated by the experience of conflict. While many questions about where in the brain RN receives information regarding the experience of conflict remain, this is the first evidence, to suggest that even at this late stage of action selection/motor output flexibility within the circuit exists, and that RN can respond and restructure behavioral actions in response to conflict.

## 4. Summary and Future Directions

### 4.1. Summary

**Figure 7:**
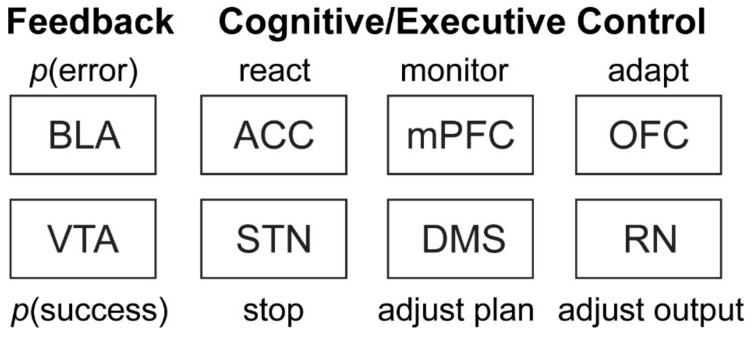
Model of stop-signal performance. Schematic representing proposed roles for ACC, BLA, DMS, mPFC, OFC, RN, subthalamic nucleus (STN), and VTA in inhibitory control. BLA and VTA work in concert to signal the probability of error (BLA) and success (VTA), respectively. This information is then used by ACC and STN to react to sudden presentation of STOP signals/experience of conflict between simultaneously active action plans. Behavioral strategies and representations of the response are monitored by mPFC and DMS, while OFC and RN make preparations to slow and adapt behavior to ensure future success.

We have presented a large body of evidence from numerous brain areas collected from rats all performing our novel variant of the stop-signal task. While this review is far from exhaustive, with other researchers examining firing in multiple other brain areas including, frontal eye field [22], supplementary eye field [150,151], striatum [152,153], substantia nigra pars reticulata [154], motor cortices [28,29,31], supplementary motor cortex [155,156], subthalamic nucleus [16,154,157,158,159], and basal forebrain [160], we have shown how DMS represents competing action sequences, and how these signals are modulated directly by ACC. ACC, unlike OFC and mPFC, has been shown to be important for detecting instances where two competing representations for actions are simultaneously active, and in turn, engaging reactive processes to shut down representation of the action represented by the first cue in rodents [13] and humans [21]. In contrast, OFC appears highly sensitive to instances of conflict [10,20], and proactively moves to slow behavior on future trials to facilitate future success. Although, understudied in the context of this task as of now, mPFC also appears to represent conflict, but does so in a delayed fashion that might be reflective of proactive strategy reassessment and updating [11]. Importantly, frontal brain areas appear to working in tandem with other non-frontal brain areas receiving information about error commission from BLA [119] as well information about the certainty of reward from VTA [14]. These processes all occur while neurons in RN begin signaling in the direction of a preferred movement [15]. On GO trials this signaling results in relatively fast automatic response, but on STOP trials this firing pattern is altered reactively to account for the addition of the alternative motor action. Remarkably, RN even demonstrates a degree of flexibility, proactively working to slow responding to the first cue on future trials after the experience of conflict (see Figure 7 for summary).

### 4.2. Future Directions

The ability to map out this circuitry from the representation of competing actions, to the detection of conflict, to motor output is made possible by the utilization of the stop-change task. Although, there are numerous variants of the stop-signal task, these findings illustrate the experimental power of using a tightly controlled and carefully designed behavioral paradigm, as well as the importance of commitment to exploring all aspects of a behavioral task. While we believe that there is much value in obtaining multiple measures of the output of brain region, as a look at the historical theoretical foundations of work in the OFC [108], ACC [161], and amygdala [116] suggest, however, the focus on a single brain region at the expense of the rest of the brain, a mindset that pervades much of neuroscience to varying degree, can ultimately limit the deeper understanding of the brain and behavior.

While our work has begun to provide a framework for thinking about inhibitory control and how it impacts the whole brain, much work is needed to investigate the complicated interplay that likely occurs between brain regions as well as to relate these findings to humans directly. To address this first issue we propose employing disruption approaches similar to the one used in our ACC lesion study [13]. With the DMS providing a physiological gauge, we can systematically assess the contributions of upstream brain regions such as OFC or mPFC to the biasing of action selection using pharmacological, pharmacokinetic, and optogenetic approaches. Similar approaches can be used to understand the contributions of these brain regions to motor output by using the physiological profile of RN as a guide, and this approach can be generalized to numerous other well-characterized and commonly used tasks of cognition.

To address relating these findings/approaches to humans, using information from animal research to inform studies in humans are necessary. This again is where using the stop-signal task, which is easily adapted into the human laboratory setting, is particularly advantageous. While important ethical considerations constrain the number of approaches available to human subject’s researchers, similar manipulations, at least in principle, can be performed in humans by taking advantage of functional magnetic resonance imaging (fMRI), electrocorticography (ECoG) [29,162], and transcranial magnetic stimulation (TMS). Ultimately understanding the degree to which findings in animal models extend to human beings is critical for validating much of the work presented here.

Finally, at the level of individual brain areas, we propose, and hope to soon add, to this work by exploring other brain areas such as BLA, cerebellum, insula, and others directly in an effort to create a profile for how the brain as whole performs the stop-signal task. The cerebellum in particular, which has direct connections with RN and seems more than likely to be involved in facilitating control, need further investigation and incorporation into existing frameworks. Moreover, understanding whether and how the circuitry underlying inhibitory control changes across the lifespan is important, as deficits in control generally, as well as specific deficits in proactive and reactive forms of inhibitory control, are common symptoms of numerous neurological and neuropsychiatric illnesses [36,114,163].

## 5. Conclusions

As neuroscientists, we remain a long way from fully understanding how or why the motorist next to me errantly bolted into the center of oncoming traffic upon the left lane receiving a green arrow. Despite this truth, our work has shown that DMS represents competing action plans and that the neural representations of these action plans are biased reactively by ACC and perhaps proactively by OFC and mPFC. Moreover, the BLA and VTA seem to provide context to frontal regions tasked with adapting behavior to changing environmental constraints, and this represent and important and, at present, understudied contribution to inhibitory control mechanisms more generally. Finally, motor areas of the brain such as RN are critical in translating changes in action plans into fully realized actions. RN in particular not only appears to bias behavior in the moment, by speeding appropriate responses on STOP trials, but also proactively by dampening movement speeds on future trials.

In short, these findings suggest that inhibitory control mechanisms encompass much more than the common frontal processes that are traditionally studied, but also highlight the need for the field to begin to expand its areas of study to include other brain areas/systems. In other words, in order to better understand inhibitory control, and other cognitive phenomenon, we need to move beyond our favored brain region(s) and commit to investigating the contributions of the brain as whole.

We are not perfect in this, as this review alone focuses just on mechanisms of detection and motor output, largely ignoring sensory input that likely plays a large role in converting diverse sensory stimuli into a common form that can be integrated and responded to. However, what we feel this review highlights is that in order to efficiently investigate the contribution of multiple brain regions to a behavior, we need a common ground, and well-established and characterized behavioral tests, amenable to physiological exploration, can provide this common space. Our hope is this review provides a guide to understanding inhibitory control as much as it provides a framework for tackling problems in systems neuroscience moving forward.

## Data Availability

Not applicable.
